# Primary cutaneous lymphoma in a patient with mastocytosis: Is there an association?

**DOI:** 10.1016/j.jdcr.2023.03.015

**Published:** 2023-04-07

**Authors:** Colin M. Kincaid, Celine Phong, Justin D. Arnold, Xiying Fan, Bonnie A. Lee, Natasha A. Mesinkovska

**Affiliations:** Department of Dermatology, University of California, Irvine, Irvine, California

**Keywords:** mastocytosis, mast cell, primary cutaneous lymphoma

Mast cells, classically known as local effector cells in allergic reactions, have become recognized in recent years for their pathogenic contributions to tumor promoting microenvironments.^1^ Patients with mastocytosis are at an increased risk for myeloid malignancies, with emerging evidence of a co-occurrence with a subset of lymphoproliferative disorders, primary cutaneous lymphomas (PCL).^2^ Because the presentation of PCL lesions can be asymptomatic and subtle, we aim to alert dermatologists of the clinical clues in patients with longstanding mastocytosis.

## Report of case

**Fig 1:**
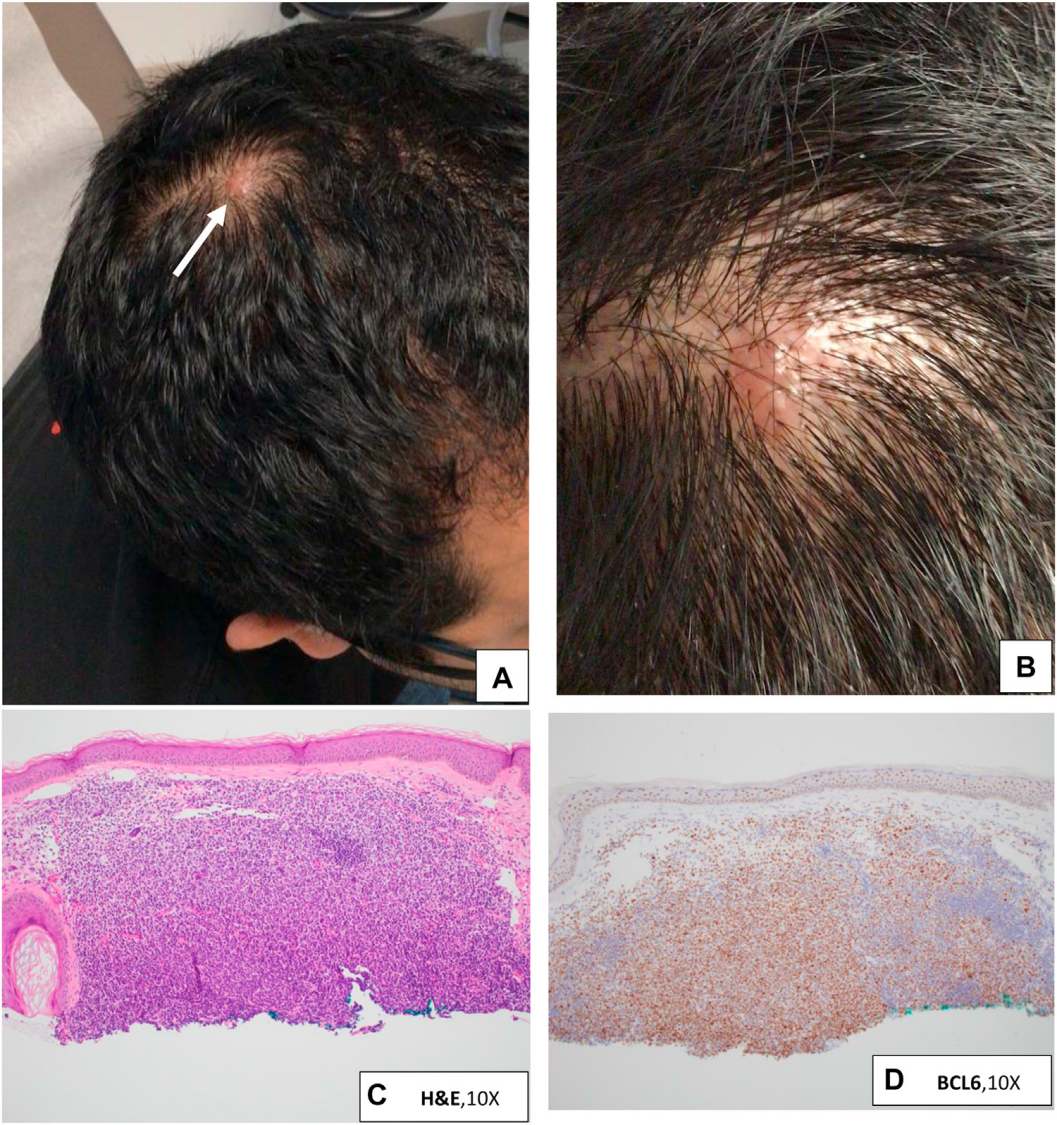
**A** (*white arrow*) and **B,** Clinical images of the asymptomatic nodule on the *right* vertex scalp in patient with mastocytosis. **C,** Hematoxylin-eosin staining of the sample (10×). **D,** BCL6 staining is diffusely positive, showing that the majority of cells present are germinal center B cells (10×).

A 35-year-old male with a 7-year history of biopsy-proven cutaneous mastocytosis, well controlled with antihistamines and dietary avoidance, presented to clinic for annual surveillance. Clinical examination revealed numerous brown macules on his trunk, consistent with urticaria pigmentosa, unchanged from prior examinations. Involving the right vertex of the scalp, he had a new 10 mm smooth, dome shaped, red-to-violaceous nodule with rare hairs (Fig 1, *A* and *B*). He had noticed the nodule slowly enlarging for 4 months and thought it was a cyst. Lymphadenopathy was not detected and the patient denied any constitutional symptoms. His recent laboratory workup, including complete blood count, serum tryptase, 24-hour urinary N-methylhistamine, and abdominal ultrasound were unremarkable. He had declined bone marrow biopsy which is part of a workup of systemic mastocytosis (SM) per World Health Organization (WHO) guidelines.^3^ The patient was instructed to return for biopsy if the lesion did not resolve within 6 to 8 weeks.

On return, the lesion was slightly more elevated. Biopsy revealed a nodular and diffuse infiltrate composed of enlarged lymphocytes with abundant cytoplasm, with aggregates of lymphocytes in the periphery (Fig 1, *C*). Immunohistochemical studies were notable for diffusely positive CD20 (cluster of differentiation 20) and BCL6 (B-cell lymphoma 6) staining (Fig 1, *D*), consistent with germinal center B cells. BCL2 was positive in a significant subset of germinal center cells. CD21 staining revealed an expanded network of follicular dendritic cells. A CD117 stain for mast cells was negative. Gene rearrangement studies confirmed the presence of a monoclonal B cell population.

A diagnosis of primary cutaneous B-cell lymphoma, follicle center type, was made. Treatment options, including triamcinolone injections, excision, and radiation were considered. The patient elected for a series of monthly intralesional triamcinolone injections (0.5 mL of 10-20 mg/mL), which resulted in improvement—but with relapses—before eventually achieving control.

## Discussion

Mastocytosis, characterized by mast cell proliferation and accumulation in the skin or other organs, is associated with hematologic neoplasm (AHN) in up to 40% of all SM cases.^3^ The classification “SM-AHN” is thus one of the main subtypes of SM defined by the WHO. Approximately 89% of SM-AHNs are associated with myeloid neoplasms that are often attributed to activating c-KIT mutations in a shared clonal progenitor.^3^ Interestingly, 10% of SM-AHN is associated with lymphoproliferative disorders. But given the distinct clonal origins of mast cells and lymphocytes, this association remains unclear.^2^

The diagnostic finding of a PCL neoplasm in this young patient with mastocytosis was surprising and we may consider it to be completely coincidental. However, mast cells can interact with B cells through the production of soluble and cell-membrane factors^4^ and can contribute to pro-tumorigenic microenvironments.^5^ In malignant neoplastic processes, mast cell derived cytokines, matrix metalloproteinases, and vascular endothelial growth factors can strongly promote proliferation, tissue remodeling, and angiogenesis, respectively.^5^ Historically, the lymphoproliferative disorders reported in association with mastocytosis have traditionally been systemic B-cell lymphomas.^2^ The question this case raises is the possibility of whether skin-residing mast cells also contribute in a similar fashion to promoting the growth of certain cutaneous neoplasms. In strong support of this, recent studies have reported the presence of mast cells, even in increased numbers, in both B- and T-cell types of PCL neoplasms. The importance for mast cells in supporting PCL tumor growth has been demonstrated in both *in-vivo* experiments and in animal models.^1^ Subsequent to this experimental evidence, there are 3 additional cases reporting a co-occurrence of PCL in patients with mastocytosis, all of which were B-cell type (Table I).^2^^,^^6^^,^^7^ In conjunction with the present case, we contemplate whether these PCL cases represent a previously underrecognized connection. While PCLs are a heterogenous group of neoplasms with varied presentation, the few described cases in patients with mastocytosis appear to have some common clinical features. They are described as new onset, asymptomatic red-to-violaceous nodules or nodular plaques, very distinct from the monomorphic, brown macules of the underlying mastocytosis. The development of the PCL tumors in affected patients (age range, 31-53 years) occurred 3 to 7 years after their initial mastocytosis diagnosis. It may be too precocious to ask dermatologists to exercise a heightened index of suspicion when evaluating novel nodular lesions in mastocytosis patients. However, we would like to alert the reader to this co-occurrence and to this growing body of evidence supporting the role of mast cells as promotors of cutaneous tumor microenvironments.

**Table I:** Summary of reported cases of primary cutaneous lymphoma in patients with mastocytosis

Author	Age (y)	Sex	Mastocytosis presentation	Time from mastocytosis presentation to PCL diagnosis	PCL presentation	PCL diagnosis
Meyer et al 2013^6^	41	M	Brown-reddish macules on trunk	3 y	Multiple bluish to purple, 2-3 cm nodular plaques on chest	Follicular B-cell lymphoma
Lee et al 2021^7^	53	M	Multiple dusky red nodules on trunk	7 y	Multiple dusky red nodules on trunk (overlap with mastocytosis presentation)	Marginal zone B-cell lymphoma
Guney et al 2022^2^	31	F	Brownish, monomorphic macules on trunk and extremities	3 y	Four reddish, 1-2 cm nodules on the upper trunk and arm	Marginal zone B-cell lymphoma
Present Case	35	M	Brown macules on trunk	7 y	Single red-to-violaceous nodule on scalp	Follicular B-cell lymphoma

*PCL*, Primary cutaneous lymphoma.

## Conflicts of interest

None disclosed.
